# WE CARE 4 KIDS: Use of a Rounding Tool in the Pediatric Intensive Care Unit

**DOI:** 10.1097/pq9.0000000000000044

**Published:** 2017-11-17

**Authors:** Rani Ganesan, Priya Rajakumar, Louis Fogg, Jean Silvestri, Jason M. Kane

**Affiliations:** From the * Department of Pediatrics, Rush University Medical Center, Chicago, Ill.; † Department of Internal Medicine, Rush University Medical Center, Chicago, Ill.; ‡ Department of Community, Services, and Mental Health Nursing, Rush University College of Nursing, Chicago, Ill.; and § Department of Pediatrics, University of Chicago Medicine and Comer Children’s Hospital, Chicago, Ill.

## Abstract

**Objective:**

To implement a daily rounding tool in a pediatric intensive care unit (PICU) to improve the discussion performance of identified clinical elements. We hypothesized that a semi-structured rounding tool created by a multidisciplinary team would be successfully implemented and sustained in the PICU.

**Patients and Methods:**

A pre–post interventional study was conducted in a multidisciplinary medical-surgical PICU. Baseline data collection of undisclosed clinical elements was performed by covert observers, which resulted in the development of a comprehensive, nurse-driven rounding checklist. Frequencies of pre- and postintervention metrics were assessed after implementation, and sustainability was assessed at 5 years.

**Results:**

Six months after implementation, 70% (7/10) of checklist elements demonstrated significant improvement. Five years after implementation, 172 of a possible 222 (74%) checklists were collected. Eighty percentage (8/10) of the measures sustained discussion frequency after 5 years of use. Nursing presence significantly improved at year 5 compared with the preimplementation period. Nursing satisfaction surveys distributed at year 5 showed that the rounding tool was useful and nurses were confident in understanding care plans at the end of rounds. Ninety-eight percentage of checklists revealed discrete transcription of qualitative daily goals.

**Conclusions:**

A semi-structured rounding tool created by a multidisciplinary team was successfully implemented, and performance was sustained at 5 years. This initiative led to improved bedside nursing presence during patient care rounds.

## Abstract 2

Supplemental Digital Content is available in the text.

## INTRODUCTION

The multidisciplinary care team model has decreased in-hospital mortality,^1^ reduced ventilator days,^2^ decreased the incidence of nosocomial infections,^3,4^ and improved patient safety.^5^ The multidisciplinary care team model encourages members from specific disciplines, including families and parents, to participate in daily patient discussions where the acute and longitudinal goals of care are delineated. Although team members are encouraged to be present and participate, actual roles and responsibilities are not well described. The success of the interdisciplinary care team model is also limited by staffing limitations that may preclude individual practitioners from participating in daily patient care rounds. Despite evidence demonstrating that multidisciplinary care team models are vital to the success of high-quality patient care, little is known about how these models of care are being adopted and sustained.

Communication failures are frequently cited as a leading cause of sentinel events reported to the Joint Commission.^6^ Efficient and safe clinical care requires providers to exchange information in a timely and accurate manner. Standardized checklists have been used to facilitate the accurate exchange of patient information between providers, patients, and families.^7–9^ These tools have been associated with lower nosocomial infection rates^4^ and reductions in overall health care costs.^10^ The use of standardized checklists may help prompt the discussion of discipline-specific patient care items in the absence of individual clinician participation during patient-centered rounds. In addition, checklists can be a reference for the medical team and family members to review when they are unable to attend daily rounds. In addition to standardized checklists, daily goals sheets have also been used to facilitate communication. Daily goals forms have improved perceived understanding of clinical goals by members of the health care team^11–13^ and have facilitated a decreased length of stay in intensive care units.^11,13^ To date, there are limited pediatric data demonstrating the sustained impact of rounding tools on the discussion of key clinical measures^14^ and the effect of rounding tools on multidisciplinary participation during daily rounds. The purpose of this study was to implement a semi-structured rounding tool to prompt discussion on prioritized patient care metrics and improve multidisciplinary participation in daily clinical rounds.

## METHODS

Using a longitudinal pre–post study design, a daily rounding worksheet was created by a multidisciplinary team and subsequently implemented. This study occurred in a 20-bed pediatric intensive care unit (PICU) with an open-care model that included medical, surgical, and congenital cardiac patients. Daily patient care rounds were directed by a PICU attending physician and typically included resident physicians, medical students, bedside nurses, respiratory therapists, clinical pharmacists, patients/parents/family members, and dieticians. A Pediatric Cardiology attending and fellow participated on daily rounds specifically for patients with cardiac disease. At 5 years, 9 PICU attending physicians and 6 Pediatric Cardiology attending physicians participated in patient rounds. This study was approved by the Rush University Institutional Review Board.

A core design team representing attending physicians, bedside nurses, nursing leadership, pharmacy, respiratory therapy, and nutrition services created a data collection tool including nationally prioritized patient-care quality metrics (ie, health care associated infections,^15^ medication reconciliation,^15^ family-centered care,^16^ and pain management^17^) that each discipline deemed vital for discussion on rounds to ensure high-quality care. The tool was organized into 8 different themes categorized by organ system, health care maintenance, diagnostic studies, discharge planning, and indwelling catheter care. A total of 35 specific items were included. In addition to specific checklist items, team members’ presence was recorded. To avoid bias, baseline data collection was performed by covert observers during patient care rounds. Observers also rated clarity of the care plan using a 5-point Likert scale. Baseline observations occurred from September 2010 to October 2010 and were specifically targeted to identify frequency of discussed items on the preintervention data collection tool.

After review of the preintervention data, the “WE CARE 4 KIDS” rounding tool (**Supplemental Digital Content 1**, http://links.lww.com/PQ9/A17) was created and corresponded to 10 themes that were inconsistently discussed or deemed necessary by the design team. All included themes were observed in the baseline data collection tool. Topics represented in the acronym included weight, extubation planning, care coordination, activities/therapies, radiographs/labs, electrolytes, parental participation, indwelling catheter prevention, drug reconciliation, and sedation/analgesia. A free-text area reserved for documentation of the delineated daily goals was also included.

The rounding tool was piloted before implementation from January through March 2011, with the senior medical resident tasked with checklist completion. Initial Plan-Do-Study-Act cycle identified challenges with checklist completion by the resident because of competing responsibilities on rounds. As a result, bedside nurses completed the checklist. Follow-up Plan-Do-Study-Act cycle resulted in charge nurse completing the worksheet or providing care if patient needs competed with bedside nursing participating on rounds. Feedback was more positive including an unintended consequence of using the worksheet to become more engaged in the rounding process. A “Nursing Time Out” was added to ensure that the all checklist items were discussed and there were no remaining questions or concerns at the completion of rounds. The final nursing statement encouraged team members to speak up before rounds concluded. Finally, nursing staff restated the goals discussed by the care team to ensure shared agreement. Completed rounding tools were maintained at bedside, and clinicians were encouraged to refer to the tool during shift-change to facilitate complete handovers of care. Worksheets were completed daily.

In the first 6 months following implementation, study investigators were present during rounds to provide real-time education and identify areas for performance improvement. At 6 months postimplementation, rounding tools were collected from 2 random weeks to assess success of implementation. Results of checklist item completion and anecdotal feedback from staff indicated successful implementation and compliance. Thereafter, worksheets were not periodically collected for formal compliance audits nor were substantive changes made to the rounding tool. During year 4 of implementation, a signature line was added for attending physicians demonstrating compliance with a system-based practice model required by institutional standards. New nursing staff were educated to “WE CARE 4 KIDS” as a part of orientation and unit on-boarding. At 5 years postintervention, sustainability data were collected from 9 random weeks and anonymous Likert-scale surveys were distributed to nursing staff assessing perceptions of rounds and the use of “WE CARE 4 KIDS.” Data from tools at 6-months and 5-years postintervention periods were analyzed. Rounding-team members were blinded to all postintervention data acquisition.

The primary outcome measure was rounding tool item completion with a secondary outcome of team member presence on rounds. Specific items analyzed included patient weight, extubation plan, care coordination, activities/therapies, diagnostic studies (including labs and radiographs), electrolytes, parental participation/communication, indwelling catheter infection prevention, drug reconciliation, and sedation plan. Items were considered completed if checked, circled, or item-specific actions were written in the space provided. Comparisons were made between preintervention (baseline) and 6-month postintervention to assess success of implementation. Sustainability was assessed by comparing 6-month to 5-year postintervention data. Checklists included at year 5 represented 9 different PICU attendings but did not reflect or describe daily census or acuity. Data were excluded if no checklist was available, or if a checklist could not be associated with a specific patient. Compliance was measured by calculating the percentage of collected checklists compared with expected. Tools were cross-referenced with daily census information to determine the number of possible checklists included in data analysis. Of note, daily census information for weekend days (Saturday and Sunday) was not readily available. The secondary outcome of team member presence was measured by comparing preintervention and at year 5.

Qualitative analysis was performed on daily patient goals documented in year 5 data. Each goal transcribed within the box designated “4 goals” was reviewed and coded using each checklist item as a theme. If the goal described one of the defined themes, the corresponding theme (i.e., checklist item) was coded as completed. The coding was compared and discrepancies were resolved through joint review by the study investigators. Checklist items and coded goals were reconciled and categorized either completed or not completed. Proportions were compared using 2-tailed Fisher’s Exact test. Weighted scores and averages were used to describe results of nursing surveys.

## RESULTS

Between September and October 2010, 66 patient rounding observations were performed to establish baseline data. Baseline data were compared to 53 patient rounding tools collected at 6 months following implementation (Table 1). There was a significant increase in the observed discussion frequency and checklist item completion of 7 of the 10 tool elements including weight, care coordination, activities/therapies, diagnostics, electrolytes, indwelling catheter/infection prevention, and drug reconciliation. At year 5, 172 checklists were collected. Ninety-eight percentage (169/172) of checklists demonstrated written daily rounding goals. A total of 769 goals were included in data analysis with an average of 4.7 goals per collected sheet. When comparing item completion between 6-month and 5-year data, 80% of the tool elements were sustained (Table 1) including extubation planning, care coordination, activities/therapies, diagnostics, parental participation/communication, indwelling catheter infection prevention, drug reconciliation, and sedation/analgesia. Compliance at 6-months was 84% (53/63) compared with 77% (172/222) at 5-years, which was not significantly different. Compared with preintervention, team member presence at 5 years revealed no change in the PICU attending, pharmacy, respiratory therapy, and dietician staff, but bedside nursing presence significantly improved (Table 2).

**Table 1.**
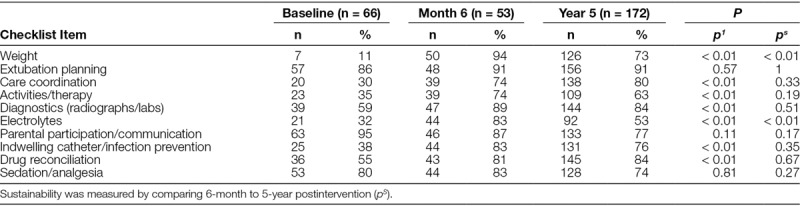
Implementation Measured by Comparing Frequency of Item Completion for Preintervention Baseline Observation Data to 6-Month Postintervention (p1)

**Table 2.**
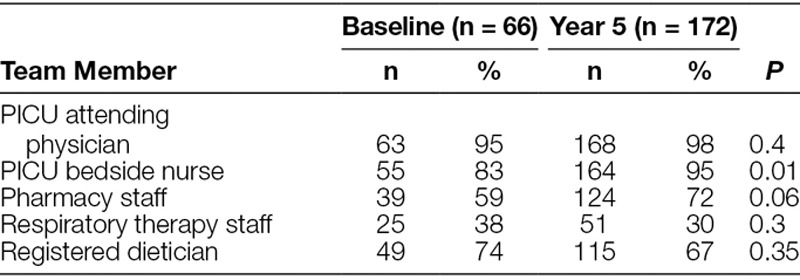
Team Member Presence Preintervention Compared with Year 5 Postintervention

A total of 36 (75% response rate) nursing satisfaction surveys were returned. Greater than 75% of respondents agreed or strongly agreed with all survey measures with 100% of nurses surveyed agreeing or strongly agreeing with the statement “I always participate in daily patient care rounds in the PICU” (Table 3).

**Table 3.**
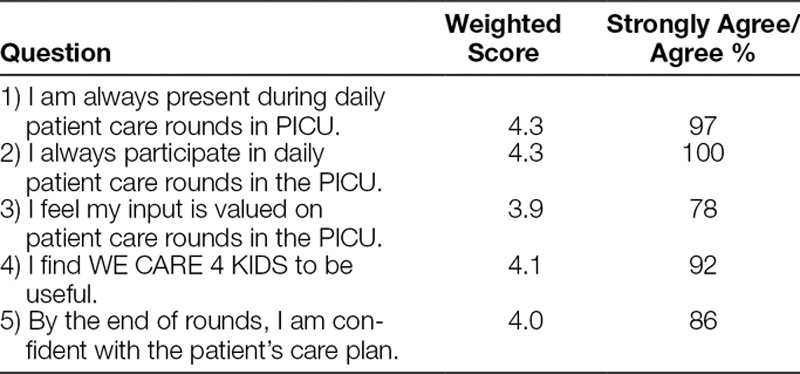
Five-Point-Likert Scale (Strongly Agree—5; Strongly Disagree—1) Assessing Nursing Perceptions of Rounds and the Use of WE CARE 4 KIDS (n = 36)

## DISCUSSION

The purpose of this study was to create and implement a sustainable rounding tool aimed at prompting discussion of important clinical measures. Although the impact of metric-specific checklists on patient clinical outcomes has been shown previously,^4,6–10^ the long-term sustainability of these tools is not well described. This study demonstrates successful implementation with improved discussion performance of 70% of critical elements and sustained improvement of 80% of elements at 5 years. The continued use of the tool improved nursing presence on daily patient care rounds. In addition, the use of WE CARE 4 KIDS has led to high nursing confidence in understanding the patient’s daily goals of care.

At 6 months, the 3 checklist items that did not show improvement were extubation planning, parental participation, and sedation/analgesia. It should be noted that these items were the highest performing during preintervention data collection. Despite initial high performance, the investigators deemed these themes vital for daily rounding discussion due to institutional priorities of family-centered care, ventilator days, and patient comfort. The high performance of these items during the preintervention period left a narrow margin for meaningful improvement. It could also be argued that the unchanged performance may indicate that the discussion prompt may not be sensitive enough to facilitate improvement. Despite the lack of change from baseline, discussion frequency of these items continued to be above 70% and sustained at 5 years. Although checklist items for weight and electrolytes did not maintain performance at 5 years, discussion frequency was significantly improved from the preintervention period and occurred more than 50% of the time.

Although the multidisciplinary team approach to care in the ICU may be considered standard of care,^18^ competing obligations may prevent providers from participation during daily rounds. WE CARE 4 KIDS improved nursing presence on rounds and resulted in a high rate of participation. The added effect of including nursing staff on daily patient care rounds is a notable strength of this study. Also, consistent participation by bedside nurses may result in opportunities to engage in patient care discussions, to participate in teaching, and to provide more informed updates to parents/families of the daily care plan. This study shows that a semi-structure rounding tool can improve the use of multidisciplinary teams, particularly nursing staff, during daily patient care rounds. Additionally, 98% of collected tools demonstrated written clinical goals and as such, an additional benefit of the tool may be the physical documentation of the stated goals of care. Satisfaction survey results at 5 years indicate that nurses felt confident in their understanding of the patient daily care plan, which was an unexpected benefit of WE CARE 4 KIDS. These perceptions may be related to the checklist completion, written transcription and restating of daily goals, or a result of improve presence during rounds.

Limitations to this study are recognized. First, item completion was used as a surrogate marker for actual discussion. As such, items may have been discussed but not marked off on the physical document or vice versa. Underestimation of item discussion frequency was addressed by reviewing and coding the daily goals, which were then reconciled with checklist item completion. It is also important to acknowledge that the study does not address the rounding tool’s impact on patient care outcomes. Although prior studies have shown an association between the use of checklists and improved patient outcomes, these findings should be interpreted with caution.^19^ Also, checklists shown to improve patient care outcomes have included discussion prompts directed at clinical practice. WE CARE 4 KIDS was designed to standardize communication themes with general discussion prompts and not to direct performance of tasks. Correlating checklist item performance to a specific metric would be out of the scope of this study. The generalizability of success of this tool may be limited by a single-PICU design. However, given the universal themes presented in the tool, a multicenter study of the checklist in its current form could reveal whether these findings can be replicated. Finally, given the relatively low number of attending physicians in the sample and absence of weekend performance, it is possible that the results were biased by specific clinician investment or weekday staffing models. It should be noted that 1 primary investigator’s service weeks was included in study samples and contributed to 15% of checklists included in data analysis at 5 years.

## CONCLUDING SUMMARY

The implementation of a structured multidisciplinary rounding tool resulted in a significant improvement in discussion frequency of key clinical elements on daily multidisciplinary patient care rounds. These data were sustained after 5 years of use. Ninety-eight percentage of patients had discrete daily goals documented. The positive impact on nursing presence and rounding culture may be the most important outcome of implementation of the WE CARE 4 KIDS rounding tool. The use of a rounding tool in the PICU can be successfully sustained and result in positive changes in rounding culture.

## DISCLOSURE

The authors have no financial interest to declare in relation to the content of this article.

## Supplementary Material
