# Supplementary material for: WE CARE 4 KIDS: Use of a Rounding Tool in the Pediatric Intensive Care Unit
Source: Pediatr Qual Saf. 2017 Nov 17;2(6):e044. doi: 10.1097/pq9.0000000000000044 (PMC6132891; doi:10.1097/pq9.0000000000000044)
Supplement: Supplementary file 1 [file pqs-2-e044-s001.pdf]

WE CARE 4 KIDS

Date: ____________________ I'm in room: __________________ ***WE*** started rounds at: _____________ ***WE*** ended rounds at: ______________

Who's here to talk about me? (Please circle)					
PICU Resident	Bedside RN	PharmD	RD	CV APN	Cards APN
PICU Attending	Charge RN	RT	Cards Attending	Cards Fellow	CV Surg Attending

Weight: NA Will weigh today
(If you are concerned that I'm not growing or if my medications need to be adjusted, please discuss weight)

Extubation Plan: NA Discussed: ______________________________________
(If I am healthy enough and don't require help from the vent, please discuss my extubation plan)

Care conference/Child Life/Dispo/DC planning: Family Support
The date of my last family care conference: ______ Dispo D/C in < 7 days - planning
(If I have been here for more than 7 days) *(If I am ready to move to another unit/ home)*

Activities/Therapies: NA Discussed: ______________________________________
(If you think I'm ready to start moving/eating and I'm not receiving tx's, please discuss if I need PT/OT/speech)

Radiographs/Labs: NA Lab schedule: ________________________________
NA Imaging schedule: ______________________________

Electrolytes: NA Discussed: ______________________________________
(Including IV electrolyte riders, electrolytes in TPN, oral electrolyte supplementation)

Kind encouragement for parents to participate: ☐ Not present, will update by: _____________
☐ Present

Indwelling catheters/infection prevention: NA Will remove: __________________
- ☐ No indwelling catheters will be removed and infection prevention has been discussed
- ☐ *(I still require frequent labs draws, CVL access, or strict monitoring of my UOP)*
- ☐ *(VAP prevention, integrity of dressing sites)*

Drug reconciliation: Discussed Drug Levels: _______________ Antibiotics: ___________

Sedation/analgesia/paralytics: NA Discussed: __________________________________
Pain score goal ________ Sedation score goal_________

NURSING TIME OUT:

The team completed **WE CARE 4 KIDS –**
Does anyone have any further questions or concerns?

☐ I understand the **4 goals** of the day to be:

1)

2)

3)

4)

Look at Dr.____'s daily progress note for my detailed plan of the day.
(Resident)

Clinical care plans were created and discussed in multi-disciplinary rounds ____
(PICU Attending)

THIS IS NOT A PART OF MY MEDICAL RECORD, PLEASE PLACE IN THE "WE CARE 4 KIDS" FOLDER LOCATED IN MY ROOM
